# Vascular Ehlers-Danlos syndrome, an often unrecognized clinical entity: a case report of a novel mutation in the *COL3A1* gene

**DOI:** 10.3325/cmj.2022.63.394

**Published:** 2022-08

**Authors:** Sanda Huljev Frković, Ana Marija Slišković, Mia Toivonen, Andrea Crkvenac Gregorek, Ana Šutalo, Majda Vrkić Kirhmajer

**Affiliations:** 1 Department of Pediatrics, University Hospital Center Zagreb, University of Zagreb, School of Medicine, Zagreb, Croatia; 2 Department of Cardiovascular Diseases, University Hospital Center Zagreb, Zagreb, Croatia; 3 Blueprint Genetics Oy, Espoo, Finland; 4 Department of Vascular Surgery, University Hospital Center Zagreb, Zagreb, Croatia; 5 Department of Cardiovascular Diseases, University Hospital Center Zagreb, University of Zagreb School of Medicine, Zagreb, Croatia; First two authors contributed equally.

## Abstract

Due to life-threatening complications, vascular Ehlers-Danlos syndrome (vEDS) is the most severe form of EDS. Because the syndrome is associated with a shortened life expectancy and variable clinical presentation, diagnosis confirmed by genetic testing is crucial to determining appropriate treatment. Despite some distinguishing features, this rare disease often goes unrecognized. Apart from surgical or endovascular treatment of serious vascular complications, medical treatment based on celiprolol helps reduce arterial complications. We report on a case of vEDS in a young man who suffered several episodes of severe vascular complications. The diagnosis of vEDS was established based on clinical manifestations and confirmed by genetic testing. A novel heterozygous pathogenic variant in the *COL3A1* gene was found. To our knowledge, this is the first case of vEDS caused by this variant.

Vascular Ehlers-Danlos syndrome (vEDS), previously named type-IV EDS, is a rare genetic disorder of connective tissue, accounting for 5% of all EDS cases (1,2). It is characterized by serious complications such as spontaneous arterial dissections, aneurysms, ruptures, and gastrointestinal perforations, which begin in early adulthood. Reported median life expectancy is 51 years (2). The syndrome is caused by heterozygous mutations in the *COL3A1* gene coding for type III procollagen, which result in the loss of mechanical strength of arteries and other hollow organs. The most common pathogenic variants in this syndrome are missense substitutions for glycine in the sequence of the collagen triple helix, and splice-site variants that lead to in-phase exon skipping (3). Herein, we report on a case of vEDS in a young man who suffered several episodes of severe vascular complications. In this patient, a novel heterozygous pathogenic variant in the *COL3A1* gene was found.

## Case report

A 23-year-old man presented to the Department of Cardiovascular Diseases of UHC Zagreb due to acute pain in the right groin area and suspected hypoperfusion of the right leg. He had a life-long history of bleeding diathesis. In early childhood, he was prone to spontaneous bruising, occasionally even presenting with hematemesis; as an infant he underwent inguinal hernioplasty. At the age of seven, he developed a duodenal hematoma and internal bleeding after a modest abdominal injury. Hematological examination ruled out hemophilia. At the age of 20, first major arterial complication occurred; he underwent urgent right-sided nephrectomy owing to spontaneous rupture of the right renal artery aneurysm. A year later, sclerotherapy was performed due to varicose veins of the left leg.

**Figure 1:**
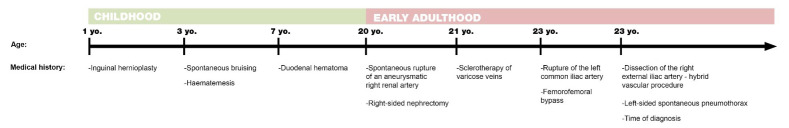
Timeline of clinical events and procedures before genetic diagnosis confirmation.

At the age of 23, a few months before admission to our institution, he was urgently operated on in a regional hospital due to a spontaneous rupture of the left common iliac artery (CIA). The artery was ligated, and femorofemoral bypass was performed. A timeline of the events is shown in Figure 1.

**Figure 2:**
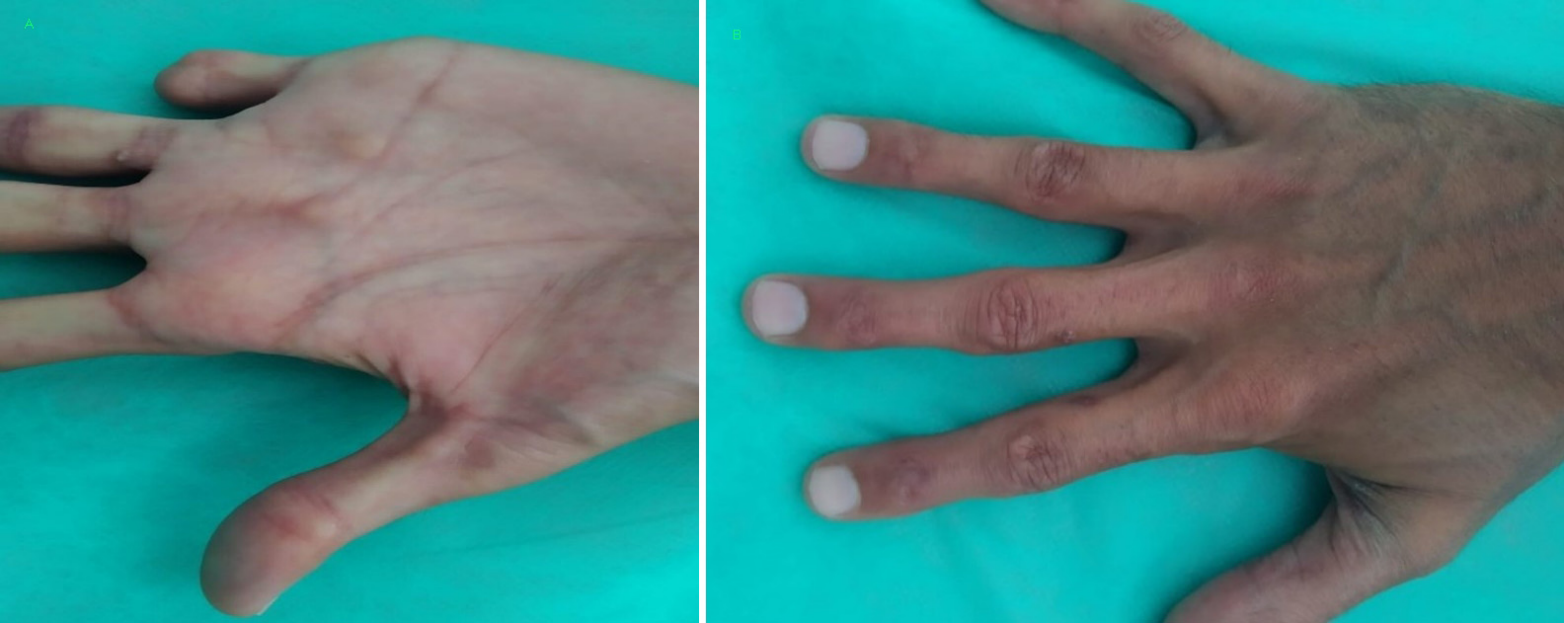
(**A**) Piezogenic papules of the palm are caused by pressure-induced herniation of subcutaneous fat through the dermis. (**B**) Acrogeria – the skin on the hands appears prematurely aged, and the subcutaneous veins are highly visible.

Physical examination upon admission revealed an asthenic constitution, thin and translucent skin with yellowish discoloration, prominent eyes, a narrow nose, thin vermilion of the lips, bilateral palmar piezogenic papules (Figure 2A), acrogeria (Figure 2B), joint hypermobility, abdominal postoperative scars, and diminished peripheral pulse in the legs. His family history was unremarkable.

**Figure 3:**
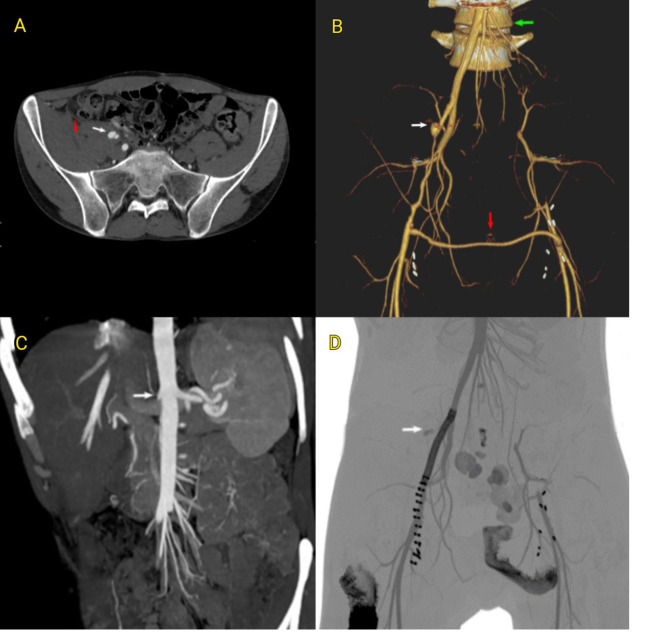
(**A**) Computed tomography (CT) angiography, axial plane, revealed a dissected right external iliac artery (EIA) with a pseudoaneurysm (white arrow) and an enlarged, hemoraginous right iliopsoas muscle (red arrow). (**B**) CT angiography, 3D Volume Render (VR) image shows a narrowed true lumen of the right EIA with a pseudoaneurysm and occluded false lumen (white arrow). Patent femorofemoral bypass (red arrow). Pelvic collaterals due to a previously ligated, ruptured left common iliac artery (green arrow). (**C**) CT angiography (maximum intensity projection) shows a stump of the right renal artery after nephrectomy due to a rupture of renal artery aneurysm. (**D**) CT angiography, VR image shows an implanted stent graft in the right EIA (white arrow). Postinterventional occlusion of the femorofemoral bypass is revealed.

Multi-slice computed-tomography (MSCT) angiography revealed a dissection of the right external iliac artery (EIA) with a pseudoaneurysm up to 14 mm in diameter and an enlarged, hemoraginous right iliopsoas muscle. The dissection extended toward the attachment of the femorofemoral bypass. The true lumen of the right EIA was patent and measured 3 to 4 mm. Due to previous vascular complications, an absence of the left CIA and right renal artery stump were observed (Figure 3A, 3B, 3C).

At a multidisciplinary team meeting, a hybrid vascular procedure was suggested. An approach through the right groin with surgical exposure of the femorofemoral crossover bypass anastomosis was used. A self-expanding heparinized stent graft was implanted into the right EIA, covering the proximal site of the dissection and pseudoaneurysm.

During the early post-procedural period, the patient developed a spontaneous left-sided pneumothorax, treated successfully with chest drainage. On the sixth post-procedural day, MSCT angiography confirmed the patency of the right EIA, with a persistent occlusion of the femorofemoral bypass (Figure 3D). Plethysmography waveforms showed a moderate left limb ischemia; the ankle brachial index was 0.55 in the left leg and 0.98 in the right leg. Considering the high risk of aorto-femoral bypass surgery and the absence of severe leg ischemia, a conservative approach was chosen.

Because a connective tissue disorder was suspected, molecular genetic testing with the EDS Panel was indicated. This panel contains genes associated with EDS and other multi-system disorders that may present with features like EDS. The analysis confirmed vEDS by identifying a novel heterozygous splice donor variant in the *COL3A1* gene: c.1149 + 2_1149 + 51del (4,5).

The patient’s medical therapy included a tolerable dose of celiprolol, a drug that may have a promising role in the reduction of vascular complications. During the four-year post-discharge follow-up, he was asymptomatic and experienced no new adverse vascular events.

## Discussion

This report aims to raise awareness about vEDS among physicians. Unfortunately, vEDS is often not diagnosed until arterial or organ rupture (1). In this case, genetic testing revealed a novel mutation of the *COL3A1* gene: c.1149 + 2_1149 + 51del, which expands the disease-causing variant spectrum. This variant has not been observed in large reference population cohorts and, to our knowledge, has not been published in the medical literature or reported in the disease-related variation databases. This variant deletes 49 bp, affects a consensus splice donor site, and leads to abnormal splicing. Variants affecting the same consensus splice site have been reported in patients with vEDS (4,5).

Our patient exhibited multiple hallmarks of the syndrome, including major criteria (spontaneous rupture of an aneurysmatic artery combined with thin skin, prominent eyes, easy bruising) and minor criteria (early-onset varicose veins and joint hypermobility) according to Villefranche (6). Despite these hallmarks, proper diagnosis was established years later, after admission to our institution. Nevertheless, because of overlapping clinical presentations with other connective tissue disorders and genetically triggered arteriopathies, genetic counseling and testing are required to support the diagnosis (2,7). A multidisciplinary approach with participation of a clinical geneticist is important for patient management.

Given the extreme tissue fragility, treatment should be conservative for as long as possible, and vascular interventions using open and endovascular approaches are only indicated in life-threatening complications, due to the possibility of further vascular trauma (8). Surgical options include arterial reconstructions, ligatures, and removal of organs with ruptured arteries, such as splenectomy or nephrectomy. Endovascular therapy includes embolization and implantation of vascular stents and endografts (9). Regarding medical treatment, a beneficial effect of celiprolol was demonstrated in a prospective, multicenter, randomized, open trial Beta-Blockers in Ehlers-Danlos Syndrome (10). Celiprolol has a unique pharmacologic profile: it is a β1-andrenoceptor antagonist with partial β2 agonist activity. A positive effect of celiprolol on collagen synthesis via the transforming growth factor-β pathway was suggested. Patients treated with celiprolol had a 3-fold reduction of arterial events in relation to untreated patients (10).

Early accurate diagnosis, genetic counseling, as well as avoiding high-risk activities and procedures are crucial in patients with vEDS.
